# Diffractive Optics From Self-Assembled DNA

**DOI:** 10.6028/jres.107.025

**Published:** 2002-08-01

**Authors:** Zachary H. Levine

**Affiliations:** National Institute of Standards and Technology, Gaithersburg, MD 20899-8410

**Keywords:** atom optics, diffractive optics, DNA, tiling, x-ray optics

## Abstract

An algorithm is presented for assembling tiles into a variable spaced grating, the one-dimensional analog of a Fresnel zone plate. The algorithm supports multi-level gratings. The x-ray properties of such a grating, assumed to be constructed from DNA are estimated, leading to the conclusion that thick structures may be useful for intermediate energy x rays, but that thin structures for soft x rays are best used as disposable masks. The diffraction of cold, coherent atoms is a plausible application for single layer stencils.

## 1. Introduction

Fresnel zone plates are used in high-spatial resolution x-ray microscopes [15,16]. The spatial resolution is determined by one half the period of the outer zones, δ*R*. The conventional notation assumes a 1:1 mark:space ratio. Just as for ordinary lenses, the spatial resolution *l* is given by *l* = *λ*/(2*NA*) where *NA* is the numerical aperture, given by *NA* = *R*/*f*, where *R* is the radius of the lens and *f* is the focal length. For Fresnel zone plates, *f* ≈ 2*RδR*/*λ* (for *R* ≫ *δR*), so for a Fresnel zone plate *l* ≈ *δR*. (The spatial resolution of a zone plate is usually quoted as the Raleigh resolution, about 1.22 *δR*. Barring advances in phase retrieval [23], improvements in the resolution of x-ray microscopes are dependent on reducing the outer zone spacing.

Such zone plates with spatial resolution below 100 nm are difficult to fabricate. For a high efficiency zone plate, it is necessary to have a significant interaction, ideally a phase shift of π or total absorption. However, x-radiation is famously penetrating. Hence, it is necessary to have a some thickness, either to create a transmission factor of order e^−1^ or less, or a phase shift of order π. The required interaction length depends on the photon energy, rising from nanometers to micrometers as the photon energy increases from the ultraviolet through 10 keV. This leads to a difficult requirement of very tall, finely spaced structures. Given the advent of highly coherent synchrotron radiation sources, improvements in the spatial resolution of x-ray zone plates would lead quickly to improvements in the resolution of x-ray microscopes.

Modern x-ray zone plates are typically made by exposing photoresist with an electron beam using technology developed to make photomasks for integrated circuits. In this paper, I put forth an admittedly speculative alternative: the fabrication of the 1D analog of a zone plate by tiling with DNA. Tiling has been shown to have the computational power of a universal Turing machine. [12] Winfree recognized the possibility of implementing computations with tiling using DNA as the tiles [32,33]. The application and adaptation of these results using DNA have been pursued by various groups, achieving addition with DNA [13], and the exclusive OR [19]. This paper explores whether it is possible and desirable to use self-assembled DNA for the fabrication of focusing x-ray optics, in particular the variable spaced grating (VSG) for one-dimensional (1D) focusing.

## 2. Diffractive Optics

Consider the Fresnel approximation to scalar diffraction theory [11]. Light is taken to propagate principally along the *z* axis. The wave function in some plane of constant *z* is determined by the wave function in the plane *z* = 0 according to

$$\begin{array}{c} \psi \left(x,y,z\right)=\frac{ke^{ikz}}{2\pi iz}e^{i\frac{k}{2z}\left(x^{2}+y^{2}\right)}\\ \int dx^{\prime }dy^{\prime }\psi \left(x^{\prime },y^{\prime };0\right)e^{i\frac{k}{2z}\left({x^{\prime }}^{2}+{y^{\prime }}^{2}\right)-i\frac{k}{z}\left(xx^{\prime }+yy^{\prime }\right)} \end{array}$$ (1)

where *x*, *y*, and *z* are Cartesian coordinates, and *k* = 2 π/λ is the wave vector of light whose wavelength is λ.

**Fig. 1:**
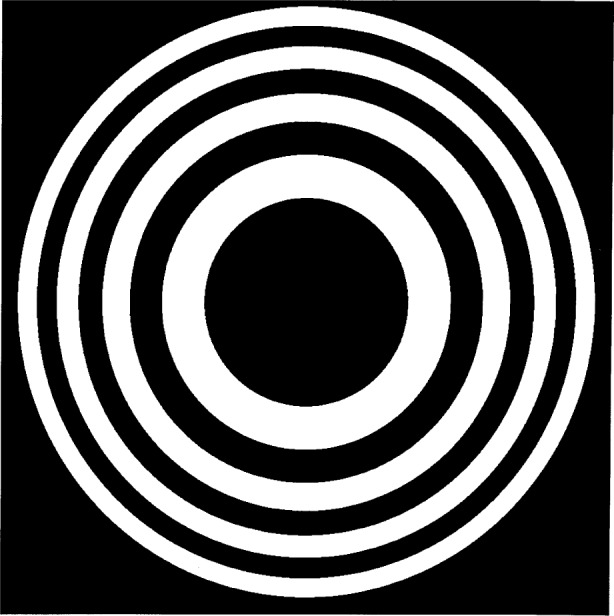
Sketch of an amplitude Fresnel zone plate.

Suppose we wish to concentrate a great deal of light from uniform illumination in the plane *z* = 0 to a point (0,0,*z*). The idea of the Fresnel zone plate arises by noting that physically eliminating out-of-phase light in the plane *z* = 0 while passing in-phase light will lead to a large value for the intensity |*ψ*(0,0,*z*)|^2^, i.e.,

$$\psi \left(0,0;z\right)=\frac{ke^{ikz}}{2\pi iz}\int_{\Delta }dx^{\prime }dy^{\prime }\psi_{0}e^{i\frac{k}{2z}\left({x^{\prime }}^{2}+{y^{\prime }}^{2}\right)}$$ (2)

where Δ is a domain of integration which corresponds to the transmissive areas of a plane with apertures and *ψ*s_0_ is the phase of the plane wave in the *z* = 0 plane. In Kirchoff’s diffraction theory, an aperture in the *z* = 0 plane is modeled simply by restricting the range of integration in the *z* = 0 plate to the transparent region. Defining the aperture by the condition $\Delta_{ZP}=\left\{\left(x,y\right)|\cos\frac{k}{2z}\left(x^{2}+y^{2}\right)+\varphi_{0}>0\right\}$, where φ_0_ is a phase, leads to a domain Δ_ZP_ consisting of set of concentric annuli, each of diminishing radius for the zones, as shown in Fig. 1. An amplitude Fresnel zone plate models this domain. The blocking material need only achieve a given thickness, sufficient to block the light; this thickness is independent of the radius. Ordinary lenses focus by achieving a constant optical path length between the image and object plane. As the physical path length may differ considerably for different rays going through a lens, this results in thick lenses. In the Fresnel lens, the optical path length differs by 2π from zone to zone. Whereas an ordinary lens must become thicker as it gets larger to accumulate a phase shift of many times 2π, a Fresnel lens has a maximum thickness. Because x rays are always absorbed in materials, the property of a maximum thickness for the zone plate is crucial for x-ray optics. Grazing incidence optics avoids the absorption problem; however, in practice the spatial resolution is limited to about 1 μm [9], vs 20 nm for Fresnel zone plates under favorable circumstances [27].

**Fig. 2:**
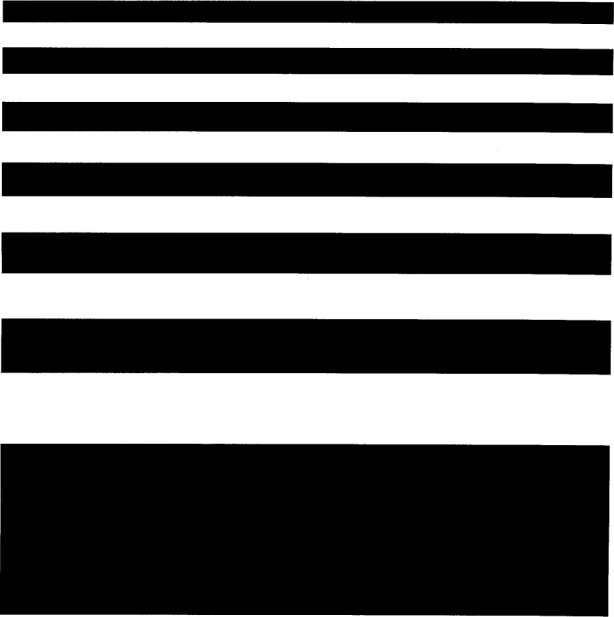
Sketch of a variable spaced grating, the 1D analog of a Fresnel zone plate.

Although the discussion here concerns focus to a point, a similar argument shows that a line focus may be obtained with an amplitude zone plate defined by $\Delta_{\text{VSG}}=\left\{\left(x,y\right)|\cos\frac{k}{2z}x^{2}>0\right\}$ where VSG stands for variable spaced grating. This domain consists of a set of variable spaced stripes, as illustrated in Fig. 2. Although Fresnel zone plates command most of the attention in x-ray optics, the 1D variable spaced grating has been recently implemented in silicon using anisotropic etching [7,8].

## 3. Algorithm for Self-Assembled Diffraction Gratings

### 3.1 Analysis of Fresnel Integral

Consider the purely mathematical problem of obtaining a variable spaced grating from self-assembled tiles. A 2D focus may be achieved by two orthogonal 1D gratings [7]; the widely-used Kirkpatrick-Baez pair implements 2D focusing with two 1D focusing elements in grazing incidence optics [14]. Such a scheme has recently been proposed for diffractive optics as well where anisotropic etching of silicon permits deep, parallel canyon-like structures [7].

In the 1D case, the aperture may be assumed to consist of a set of stripes parallel to the *x* axis. Further, the stripes may be pixellated, i.e., the stripes have widths which are are integer multiples of some distance *δ*. I wish to consider using self-assembled tiles, which could be implemented with DNA, to create a variable space diffraction grating along the *y* axis. The grating itself will be created by extending the pattern orthogonally parallel to the *x* axis with simple repetition. The construction will be of a pixellated version of a variable spaced grating: each pixel will be transparent or opaque to maximize intensity at the focus but, in contrast to the continuous case, all zones will be integer multiples of some minimum size. Such a constraint is necessary for the construction to be a tiling. Mathematically, we wish to construct a set *S*(*δ*, *α*, *φ*_0_) of positive integers *n* such that *I_n_*(*δ*, *α*, *φ*_0_) > 0 where

$$I_{n}=\frac{1}{\delta }\int_{\left(n-\frac{1}{2}\right)\delta }^{\left(n+\frac{1}{2}\right)\delta }dy\;\cos\left(\alpha y^{2}+\varphi_{0}\right)$$ (3)

where $\alpha =\frac{k}{2z}=\frac{\pi }{\lambda z}$. While this integral has an analytic form in terms of the Fresnel integrals, a simple approximation is more useful in this context.

$$I_{n}\approx \cos\left(n^{2}\alpha \delta^{2}+\varphi_{1}\right)$$ (4)

is sufficient to ensure the correct sign as long as $\left(n+\frac{1}{8}\right)\alpha \delta^{2}\leq \pi$, which is a very weak restriction from a practical point of view: the ideal zone spacing must not be smaller than *δ*. Here, $\varphi_{1}=\varphi_{0}+\frac{1}{8}\alpha \delta^{2}$.

Using the result of Eq. (4), for *n* not too large, it is sufficient to construct the set

$$S=\left\{n|\left(n^{2}\alpha \delta^{2}+\varphi_{2}\right)\;\mathrm{mod}\;2\pi <\pi \right\}$$ (5)

where $\varphi_{2}=\varphi_{1}+\frac{\pi }{2}$. Let $M=\frac{2\pi }{\alpha \delta^{2}}$ be an integer. (For a given *λ* and *δ* this condition may be achieved by a suitable choice of *z*.) Then,

$$S=\left\{n|\left(n^{2}+p\right)\;\mathrm{mod}\;M<\frac{M}{2}\right\},$$ (6)

where *p* = *φ*_2_/(*αδ*^2^). (One may choose *p* freely because it depends linearly on *φ*_0_ which may be chosen freely.)

### 3.2 Tiling Construction for Variable Spaced Grating

Consider a 2 × *N* array of tiles named *A_n_* and *B_n_*, *n* = 0,…,*N* − 1. The tile edges will be denoted by N, ℰ, W, and S for north, east, west, and south, respectively. Under the rules of the tiling the S of *A_n_*_+1_ and *B_n_*_+1_ must be the same as the N edge of *A_n_* and *B_n_*, respectively. Furthermore, the ℰ edge of *A_n_* must be the same as the W edge of *B_n_*. The tiles *B_n_* are given by

 (2n+1)modM (2n−1)modMBn2 (2n−1)modM 

and the tiles *A_n_* are given by

**Fig. 3:**
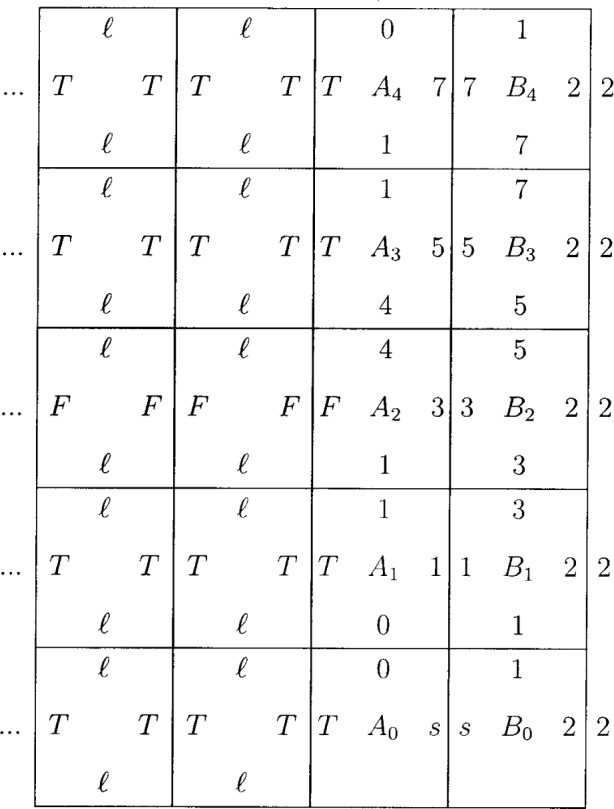
Example of tiling for a variable space grating for *M* = 8 and *p* = 0. *A*_0_ and *B*_0_ are starting tiles. A column of 2’s to form a vertical frame is also assumed. The ellipses indicate tiles which are repeated indefinitely to form stripes. The symbols *T*, *F*, *ℓ*, *s*, and the numbers are distinct symbols.

 n2modM (n2+p)modM<M/2An(2n−1)modM (n−1)2modM  (7)

for *n ≥* 1. The N edge of each tile is the sum (mod *M*) of the ℰ and S edges. The tiles *A*_0_ and *B*_0_ are starting tiles and are slightly modified. Their S edge is left blank (it is not involved in the tiling) and a special symbol *s* links these two uniquely as seen in Fig. 3. The starting conditions also assume a long straight border of tiles labeled 2 on their W side.

The meaning of (*n*^2^ + *p*) mod *M* < *M*/2 is that it is *T* if true and *F* if false. The tiling of the *B_n_* is uniquely determined because the presence of the label 2 leads, for *M* even, to only *B* tiles being placed against the column of 2’s and nowhere else; moreover, there is only one tile with a given S edge, so the N edge of *B*_0_ determines the tiling of all the *B_n_*. The tiling of the *A_n_*_+1_ is also uniquely determined: the N side of *A_n_* and the W side of *B_n_*_+1_ are sufficient to identify a unique tile. Note that there are not more than *M* distinct *B* tiles and *M*^2^ distinct A tiles. To construct the grating, these tiles must be augmented by the tiles *T* and *F* labeled by

 ℓ T T ℓ and ℓ F F ℓ  (8)

respectively, where ℓ is a new symbol.

**Fig. 4:**
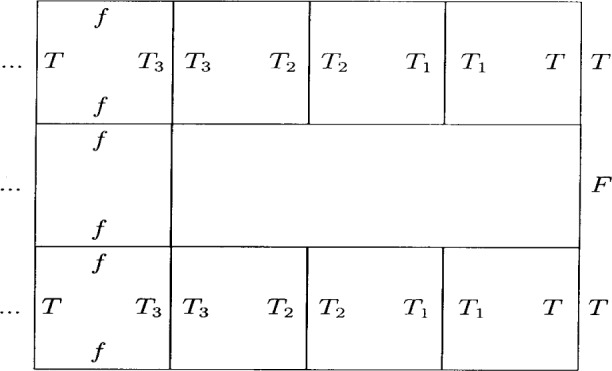
Example of implementation of stripes for a grating as a stencil for the case *q* = 4. The bridging tiles are for mechanical strength. The grating contains voids.

The tiles *T* and *F* propagate stripes whose type is determined from the label on W the side of each *A_n_*. The *A_n_* with a *T* label define the *n* in the set *S* of Eq. (6). These tiles construct a variable space grating within a quarter plane. An example is given in Fig. 3. No attempt is made to confine the construction to a finite size, but this could be done externally (e.g., by providing a finite area for the growth of the pattern). Achieving a practical diffraction efficiency in the case of an x-ray grating (at least 1 %) will require the tiling to be extended upward along the *z* axis as discussed below. For the case of atom diffraction, a stencil with clear apertures is required. Such a diffraction grating may be implemented by generalizing the tile *T* to *T*_1_, …, *T_q_*, not propagating *F*, and adding a bridge tile *f*, as shown in Fig. 4.

The construction of the tiles *A_n_*, *B_n_*, *T*, and *F* is not addressed explicitly in this paper. However, key requirements have been demonstrated previously, specifically binary addition [13] and signal threads [26].

The number of distinct tiles may be reduced by noting that *B_n_* may be implemented by having it add 2 to its S side mod *M* to get its N side. If *M* is a power of 2, this is particularly simple to implement. Transferring the S side to the W side is a relatively routine use of signal threads [26]. Similarly, *A_n_* may be implemented by adding its S side to its ℰ side mod *M*. The logical comparison required for the W side of *A_n_* simply requires reading the most significant bit of the N side, if *p* = 0 or *p* = *M*/2 and *M* is a power of 2. Hence, the number of distinct tiles required is comparable to that needed to implement addition modulo a power of 2 [13].

Multi-level diffraction gratings [10] may be achieved within this scheme as well. The tiles *A_n_* would have their W side labeled by (*n*^2^ + *p*) mod *M*, and the pair of tiles *T* and *F* would become a set of *M* tiles each with the appropriate height. The efficiency of multi-level diffraction gratings can be much higher than two-level ones.

In principle, the spatial resolution of the grating can be determined by the 2 nm diameter of DNA, even if it takes several steps to perform the computation of the *A_n_* and *B_n_*. The branched tiles (*T* or *F*) need not be orthogonal to direction N. The smaller the angle from N the smaller the pitch.

It is not immediately obvious how to turn this construction into a Fresnel zone plate, although if high efficiency can be achieved, two 1D variable spaced gratings can be effective for 2D focusing [7]. However, all zones of a Fresnel zone plate have equal area. Hence, one could imagine constructing a binary amplitude Fresnel zone plate by having *M* tiles which combine in a cyclic topology in 1D (i.e., counting from 0 to *M* − 1) but which are arranged in a spiral in 2D. Assuming the DNA could bend over a range of angles and would bond covalently without guidance from sticky ends (exposed bases) such a construction could be attempted. It is sufficient to construct an annulus of the zone plate, as the central region is usually blocked in practice.

## 4. DNA as a Material For X-Ray and Atom Optics

The x-ray properties of any material depend principally on the chemical composition and density. The stoichiometry of standard DNA is given in Table 1. The variation due to the composition of the base pairs is negligible. Also neglected here is the difference in composition between standard DNA and cross-linked DNA. The density of DNA may be estimated from the properties of an isolated double helix. The diameter is 2 nm; a base pair has a length of 0.34 nm [18]. Assuming DNA is packed in a simple square lattice in two dimensions, a base pair has a volume of 1.3 nm^3^. The molecular weight of one pair, one-half of H_51_C_39_N_15_O_24_P_4_, is 618.902 amu [5] or 1.03 × 10^−21^ g. Hence, the density in this estimate is 0.8 g/cm^3^. In practice, this is likely to be an upper bound, as DNA need not pack densely.

**Fig. 5:**
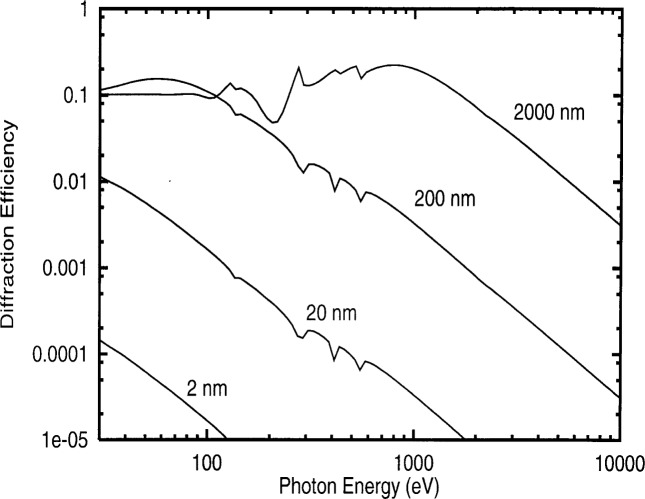
Efficiency of diffraction grating composed of 1, 10, 100, and 1000 layers of DNA double helices at 2 nm per layer. A density of 0.8 g/cm^3^, chemical composition of H_51_C_39_N_15_O_24_P_4_, and mark-to-space ratio of 1:1 are assumed. Calculation performed using Ref. [1].

Using the density and composition parameters, it is possible to get a quick estimate of the diffraction efficiency of a transmission grating, as shown in Fig. 5. To achieve a reasonable efficiency, many hundreds of layers of DNA molecules will be required. The efficiency could be improved markedly through a multi-level structure in which the height is varied to achieve an approximate quadratic phase factor.

Radiation damage is an issue for these structures. With soft x rays, samples may be imaged tomographically with some damage for dosages of 24 MGy [16,31], which is equivalent to 150 eV of absorbed radiation per base pair. Such damage could limit the useful lifetime of a DNA diffraction grating to perhaps an hour. The estimate may be unduly pessimistic: the cross-linked nature of the DNA may increase the radiation resistance. The application of ligase to increase the covalent bonding (and introduce relatively heavy P atoms) is helpful in this context [34]. The use of cryogenics has been shown to improve the threshold for morphological damage while the specimen is frozen to 10 GGy [21,30]. Moreover, even if the structures were susceptible to radiation damage, it is possible they could be used as masks (used several times) to expose substrates with very fine patterns.

Recently, atom optics have been used to explore the possibility of lithography with resolutions in the few nm regime [2]. The observation of Bose-Einstein condensation in atomic traps [6] has also led to increased recent interest in atom optics, e.g., to study the momentum distribution and coherence of the condensates [17,28]. Although most manipulation of atoms is based on electromagnetic (including optical) interactions [22], microfabricated atom optics have been developed as well [3]. In particular, Fresnel zone plate stencils with an outer zone spacing of 50 nm, suitable for the diffraction of atoms, have been reported recently [25], representing an improvement on outer zone spacings of 415 nm [4] and 230 nm [29] achieved a decade ago. The advantages of material optics over their electromagnetic counterparts are ease of use and independence of the atomic species diffracted by the optic [4].

A variable spaced transmission grating made out of a single layer of DNA could serve as a diffraction grating for cold atoms, such as those found in a Bose-Einstein condensate [6]. The thermal de Broglie wavelength of a particle at temperature *T* is given by

$$\lambda =\frac{2\pi \hbar c}{{\left(2Mc^{2}kT\right)}^{1/2}}$$ (9)

where *M* is the mass of the particle, *k* is Boltzman’s constant, and *T* is the temperature. For the case of Rb atoms at 100 nK, λ = 1.1 μm. This length may be compared to the 2 μm ×8 μm size achieved for self-assembled DNA films [33]. Moreover, self-assembled DNA with holes of controlled size of 10 nm to 20 nm have been realized, [20] a key feature of the technology considered here. Free standing carbon films 3 nm to 4 nm thick and 75 μm square containing large holes are a low-cost commercial product^1^ [24] suggesting that the requirement for a free standing film will not be too onerous.

To assemble a diffraction grating, a high degree of accuracy in placement is required. Improving the rigidity of DNA for use in molecular electronics is a current research topic [34].

## 5. Conclusions

An algorithm for constructing 1D diffractive optics using tiling has been presented. The algorithm may find practical application using tiles constructed from artificial DNA. In principle, ultra-fine resolution diffractive optics may be created with this method. A single layer would suffice to focus coherent condensed atoms to a line. A multiple layered structure could find application either as an x-ray photomask or directly, although radiation damage is a key issue.

## Figures and Tables

**Table 1:** DNA stoichiometry [18] sums over adenine (A) and thymine (T) as well as cytosine (C) and guanine (G) are given because these molecules are paired. The values are given for the constituent molecules bound in DNA. The total figure is for one of each base, four deoxyribose molecules and four phosphate bonds. Because the AT and CG pairs are so similar the x-ray absorption properties will depend negligibly on the detailed composition, as shown in the lines “All AT” and “All CG”.

	H	C	N	O	P
Adenine + Thymine	9	10	7	2	0
Cytosine + Guanine	10	9	8	2	0
Deoxyribose	7	5	0	1	0
Phosphate bond	1	0	0	4	1
Total	51	39	15	24	4
All AT	50	40	14	24	4
All CG	52	38	16	24	4

## Footnotes

1 Certain commercial equipment, instruments, or materials are identified in this paper to foster understanding. Such identification does not imply recommendation or endorsement by the National Institute of Standards and Technology, nor does it imply that the materials or equipment identified are necessarily the best available for the purpose.
